# Burdensomeness and fear of pain in adolescents with suicidal ideations and attempts

**DOI:** 10.1192/j.eurpsy.2023.481

**Published:** 2023-07-19

**Authors:** L. Esina, E. Rasskazova, V. Sadovnichaja

**Affiliations:** ^1^Clinical Psyhology Department, Moscow State University; ^2^Clinical Psyhology Department, Moscow State University, Mental Health Research Center, Moscow, Russian Federation

## Abstract

**Introduction:**

Relationship to physical pain (Joiner, 2005, O’Connor, Kirtley, 2018, Galynker, 2017) and psychological pain (Eisenberger et al., 2003) are related to the risk of suicidality in adolescents.

**Objectives:**

The aim was to reveal the relationship between interpersonal needs, relation to pain and suicidality in adolescent with or without suicidal thought and/or attempts.

**Methods:**

92 adolescents without suicidal thoughts (16 males, 12-21 years old), 132 adolescents with experience of suicidal thoughts in the past or present (22 males, 12-21 years old) and 55 adolescents (7 males, 12-21 years old) ongoing clinical treatment due to suicidal actions or attempts filled Interpersonal Needs Questionnaire (Van Orden et al., 2012), Discomfort Intolerance Scale (Schmidt et al., 2006), The Pain Catastrophizing Scale (Sullivan et al., 1995).

**Results:**

Adolescents with suicidal actions score lower in the fear of physical pain (χ²=18.19, p<.01) but higher in powerlessness (χ²=6.58, p<.05). They also experience higher burdensome and thwarted belongingness (χ²=34.50-87.92, p<.01). Their burdensome is more related to avoidance of pain (r=-.38 comparing to r=.06 and r=.04 in the control groups) while fear of pain is related to burdensome in the two control groups only.

**Conclusions:**

In adolescents with suicidal actions their avoidance of pain could be the target of psychotherapy while in adolescent with suicidal thoughts or without it there is a fear of pain.

Study is supported by Russian Science Foundation, project 22-28-01524.

**Disclosure of Interest:**

None Declared

